# PiDNA: predicting protein–DNA interactions with structural models

**DOI:** 10.1093/nar/gkt388

**Published:** 2013-05-22

**Authors:** Chih-Kang Lin, Chien-Yu Chen

**Affiliations:** ^1^Center for Systems Biology, National Taiwan University, Taipei 106, Taiwan and ^2^Department of Bio-Industrial Mechatronics Engineering, National Taiwan University, Taipei 106, Taiwan

## Abstract

Predicting binding sites of a transcription factor in the genome is an important, but challenging, issue in studying gene regulation. In the past decade, a large number of protein–DNA co-crystallized structures available in the Protein Data Bank have facilitated the understanding of interacting mechanisms between transcription factors and their binding sites. Recent studies have shown that both physics-based and knowledge-based potential functions can be applied to protein–DNA complex structures to deliver position weight matrices (PWMs) that are consistent with the experimental data. To further use the available structural models, the proposed Web server, PiDNA, aims at first constructing reliable PWMs by applying an atomic-level knowledge-based scoring function on numerous *in silico* mutated complex structures, and then using the PWM constructed by the structure models with small energy changes to predict the interaction between proteins and DNA sequences. With PiDNA, the users can easily predict the relative preference of all the DNA sequences with limited mutations from the native sequence co-crystallized in the model in a single run. More predictions on sequences with unlimited mutations can be realized by additional requests or file uploading. Three types of information can be downloaded after prediction: (i) the ranked list of mutated sequences, (ii) the PWM constructed by the favourable mutated structures, and (iii) any mutated protein–DNA complex structure models specified by the user. This study first shows that the constructed PWMs are similar to the annotated PWMs collected from databases or literature. Second, the prediction accuracy of PiDNA in detecting relatively high-specificity sites is evaluated by comparing the ranked lists against *in vitro* experiments from protein-binding microarrays. Finally, PiDNA is shown to be able to select the experimentally validated binding sites from 10 000 random sites with high accuracy. With PiDNA, the users can design biological experiments based on the predicted sequence specificity and/or request mutated structure models for further protein design. As well, it is expected that PiDNA can be incorporated with chromatin immunoprecipitation data to refine large-scale inference of *in vivo* protein–DNA interactions. PiDNA is available at: http://dna.bime.ntu.edu.tw/pidna.

## INTRODUCTION

Interactions between transcription factors (TFs) and their binding sites play important roles in many biological processes. Many previous studies have attempted to characterize the binding sequences of a TF by summarizing its known sites as a position weight matrix (PWM), and then using the PWM to discover more potential binding sites in the genome. However, the number of well-characterized PWMs is still far behind the number of known TFs. In this regard, it is desirable to exploit other resources, such as protein–DNA complexes in protein structure databases, to improve the coverage of TFs on which the prediction of binding sites can be made or improved.

In the past decade, as more protein–DNA complexes are becoming available, researchers are able to investigate protein–DNA interactions at an atomic level (1,2). Many studies have developed structure-based computational methods for predicting protein–DNA interactions (3–5). Most of the studies focus on predicting protein functions or DNA-binding residues based on structure models (4–8), and the prediction of binding residues has achieved a high degree of accuracy (6,8). Many potential functions, including physics-based and knowledge-based (7,9–11), have been developed for improving protein–DNA docking (11–13). These potential functions are also being applied to predict binding specificity and construct PWMs, as long as a complex structure exists (9,14–18). A recently published approach further uses an energy function that is uniquely trained on each structure for recognition of TF-binding sites (19). While the prediction of PWMs based on native structures achieves better performance year after year, this success has also been extended to synthetic protein–DNA complexes in our recent study (15).

To further use the available structural models, the proposed Web server, PiDNA, aims at first constructing reliable PWMs by applying an atomic-level knowledge-based scoring function on numerous *in silico* mutated complex structures, and then predicting the interaction between a protein and a single DNA sequence using the PWM suggested by the structure models with small energy changes. Given a protein–DNA complex structure, all of the potential DNA sequences with limited mutations from the native sequence in the co-crystallized structure are scored by the atomic-level knowledge-based scoring function, and a subset of relatively high-specificity sequences is selected to construct a PWM for re-ranking the mutated sequences and for making more predictions. In addition to a ranked list that reveals the relative binding specificity of the mutated sequences, users can also download mutated protein–DNA complex structures of interest for other applications.

Although many Web servers exist for predicting protein–DNA interactions based on structure models, most of them are designed to predict whether a protein binds to DNA such as iDBPs (20) or to predict DNA-binding residues such as DISPLAR (21), DNABINDPROT (22) and PreDNA (8). Moreover, the Robetta server allows for residue design on DNA-binding proteins by modelling the changes in binding-free energy associated with amino acid and base substitutions, given a protein–DNA complex (23). On the other hand, Web servers that use protein–DNA complexes to predict PWMs or sequence specificity of TFs are still limited. Our previous work, DBD2BS, was developed for predicting PWMs from protein unbound structures (24). A more similar work to PiDNA is a recently published Web server, 3DTF, which also uses knowledge-based potential on mutated structures to produce PWMs (25). The 3D-footprint database also provides pre-calculated PWMs for all protein–DNA complexes in the RCSB Protein Data Bank (PDB) (26), where the PWMs are constructed by using a hybrid approach that combines contact and readout models (27). In this regard, both 3DTF and 3D-footprint are included in this study for comparison when evaluating the performance of PiDNA.

## WEB INTERFACE

### Input

#### Step 1. Provide a structure

A protein–DNA complex is expected as the input to PiDNA. For the user’s convenience, PiDNA has collected all of the existing protein–DNA complexes deposited in PDB on the local site. In the 28 November 2012 release, there are 2589 structures that contain both protein and DNA molecules. The four-character PDB identifiers can be specified in this step. Alternatively, users can upload their own structures for analysis.

#### Step 2. Select a double-stranded DNA

After a structure is given, PiDNA automatically detects the chain identifiers of double-stranded DNA (dsDNA) molecules present in the structure. If only one dsDNA is found, PiDNA will use it by default for mutation analysis. On the other hand, if more than one dsDNA is detected, PiDNA will provide relevant information and wait for the user to select one dsDNA to make predictions. For example, the PDB structure ‘1RUN’ contains two dsDNA units, where chain C is paired with chain F (denoted as ‘C <-> F’) and chain D is paired with chain E. As long as the dsDNA for analysis has been determined, PiDNA will provide the information about the paired complementary bases. Bases within 4.5 Å with respect to any heavy atoms of a protein chain are highlighted in red, where the threshold of 4.5 follows the suggestion from previous studies (5,9). The contact residues along with the protein chain identifiers appear when the mouse moves over a contact base. At this stage, the user is allowed to specify the range of base pairs to mutate as well as the maximum number of mutations. Users are suggested to start with a small value on the setting of the maximal number of mutations, especially for a small binding site. If the number of mutations is large with respect to the site length, the assumption of the rigidity of the DNA backbone might not hold anymore.

### Output

After clicking the ‘submit’ button in Step 2, PiDNA synthesizes the structures for all sequence combinations with limited mutations from the native sequence. As exemplified in Supplementary Table S1, a position has three potential bases to mutate to. In this regard, a DNA sequence of length six can be mutated into 153 mutant forms when up to two mutations are allowed. For each mutated sequence, the corresponding synthetic structure is generated (see Materials and Methods) and the change in binding free energy is estimated (see Materials and Methods). In the result panel, only partial mutated sequences are listed, whereas the complete list can be downloaded as a text file. The list can be ranked by different columns: the number of mutated positions, the estimated change in binding free energy, the final prediction score (see Materials and Methods) and the root-mean-square deviation (RMSD) with respect to the native structure. In addition to the ranked list of the sequences with limited mutations, PiDNA also reports a position frequency matrix (PFM) constructed from structural models with small energy changes. With the reported PFM, the following three steps can be selected for execution:

#### Step 3a (optional). More predictions on manually modified sequences

More predictions can be made based on the PFM constructed previously. At this stage, the user can also select a mutated sequence to generate the structure for visualization and downloading. The mutated sequence can be further modified manually. Users will need the JAVA Runtime Environment to activate the Jmol applet (http://jmol.sourceforge.net/).

#### Step 3b (optional). More predictions on sequences set by uploading

At this stage, the user can make more predictions by uploading files. The sequence file can be prepared according to an example file provided by the server.

#### Step 3c (optional). More predictions on random sequences generated by the server

At this stage, the user can make more predictions on a larger set of sequences generated by PiDNA.

## MATERIALS AND METHODS

### Base mutation

To perform base-pair mutations on a given structure, structure models for different base types are required in advance. The atom coordinates of a particular base (A, T, C or G) are collected from the available PDB structures that contain dsDNA. The coordinates retrieved from a single base are considered as a rigid body and structurally aligned with the others. In total, 10 000 structure models are collected for each of the base types, and the averaged atom coordinates are stored as the template model for each base type. Whenever a base pair, e.g. A <-> T, is requested to mutate into another one, e.g. C <-> G, the base ‘A’ is replaced by the template model of ‘C’ and the base ‘T’ is replaced by the template model of ‘G’ without affecting the backbone structure of the dsDNA. For example, the replacement of base ‘A’ by base ‘C’ is performed by the following procedures: (i) consider the template model of ‘C’ as a rigid body and superimpose the coordinates of the atom ‘N1’ in ‘C’ onto the corresponding atom ‘N9’ in ‘A’, (ii) align the normal vector of the new base plane with that of the original base plane, (iii) align the interior bisector of a specified angle in ‘C’ with that of ‘A’, and (iv) remove the atom coordinates of base ‘A’ from the complex structure. The information regarding the atom names and the atoms selected to construct the base planes and angle bisectors is provided in Supplementary Figure S1.

### All-atom scoring function

PiDNA uses an all-atom distance-dependant knowledge-based scoring function to evaluate the preference of the synthetic complexes with respect to the native structure. Let atom *i* be an atom from the protein chains in a complex structure, and atom *j* be an atom from the DNA chains. An atom pair *i* and *j*, with a distance of *r*, is scored by the following equation:

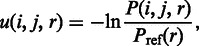

where *P*(*i*, *j*, *r*) = *N*_obs_(*i*, *j*, *r*)/Σ*_r_N*_obs_(*i*, *j*, *r*) and *P*_ref_(*r*) is a weight function that reduces the influence of a long distance, as a longer distance covers more atom pairs. All the combinations of the frequency *N*_obs_(*i*, *j*, *r*) are derived from a pre-collected protein–DNA complex set, where *i* can be any atom type from amino acids and *j* can be any atom type from nucleotides. The α-carbon in amino acid cysteine is an atom type different from the α-carbon of alanine. In total, there are 167 atom types from proteins and 82 atom types from DNA. More details about the weight function *P*_ref_(*r*) and how the table entries *N*_obs_(*i*, *j*, *r*) are constructed based on the protein–DNA complex database can be found in our previous study (15).

For a given complex, the binding free energy, Δ*G*, is defined as the sum of all the statistical potential of the observed atom pairs (9):

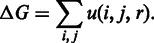



Whenever a mutated structure is generated, PiDNA re-calculates the Δ*G* and denotes it as Δ*G*′. Afterwards, all mutated structures along with the native one are sorted by the change of Δ*G*, i.e. Δ*G*′ − Δ*G*_native_, in ascending order. The minimum value of Δ*G*′ suggests acceptable flexibility on the structure change. In this regard, PiDNA discards the mutated structures with a change of Δ*G* larger than the absolute value of Δ*G*′_minimum_ − Δ*G*_native_. In other words, although a negative change on Δ*G* is preferred, a positive change that falls in the range of acceptable flexibility is still favourable.

With the mutated structures that satisfy the flexibility criterion, PiDNA uses the corresponding mutated sequences to construct a PFM. PiDNA will then use the derived PFM to re-rank the binding sites by the procedure described in the following section. The analyses shown in this study reveal that the PFM scoring is, in general, superior to the scoring based on the estimated energy change when the number of mutations is getting larger. This might be owing to the fact that PiDNA assumes that the backbone of the dsDNA molecule does not suffer conformational change when base-pair mutations are performed, but this assumption loses its validity when the number of mutations increases. PiDNA also provides the information regarding potential structural change by calculating the RMSD of the mutated structure against the native complex as follows:

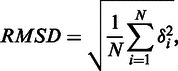

where *δ* is the distance between *N* pairs of equivalent atoms.

### PFM scoring

Given a PFM, *P*, with a width of *w*, a particular sequence *S* is scored with the following equation after proper alignment (given that the *k*-th position of *S*, denoted as *S_k_*, is aligned with the first position of the PFM without insertions or deletions and assuming that the length of *S* is larger than or equal to *w*):

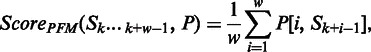

where the first dimension of the PFM, *P*, is the position identifier and the second dimension specifies the type of bases: A, T, C or G. In case the length of the substring starting from *S_k_* is smaller than *w*, the value of *w* is adjusted to a proper value before calculating the scores.

The *Score_PFM_* is assigned as the final score to reflect the relative sequence specificity of the mutated sequences. The performance of PiDNA in distinguishing the high-specificity sites from the lower ones is evaluated in the following section. In PiDNA, the PFM is calculated from aligned mutated sequences with equal length. Therefore, *k* is always set to 1 when calculating *Score_PFM_*. On the other hand, to generate *Score_PFM_* from an annotated PFM or from the PFM predicted by existing Web servers for comparison, the value of *k* is set to a proper value after sequence alignment with consideration for the reverse complementary form.

## EVALUATION AND DISCUSSION

### Validation sets

This study first evaluates whether the PFMs constructed using the highly reliable structures with limited mutations are consistent with the known binding sites of the query protein. Mouse and yeast TFs with structure models available in PDB are examined to see if annotated PFMs can be found in literature or databases. In this study, PFMs are collected from the study of Morozov *et al.* (9), TRANSFAC 7.0 Public 2005 database (28) and MYBS (29). This results in 30 proteins for evaluating the PFM quality, denoted as validation set 1 (Supplementary Table S2).

To further evaluate the performance of PiDNA in distinguishing high-specificity sites from lower ones, this study uses *in vitro* protein-binding microarrays (PBMs) to retrieve relative specificity information of a DNA-binding protein against different dsDNA sequences. The PBM data provide information regarding how strongly a given protein binds to a probe relative to the others. The mouse and yeast proteins in the UniPROBE database (30) are examined against validation set 1 for available structures. The available PDB structures are further examined to remove structures in which the binding sites are bound by hetero-multimeric proteins. Because not all lengths of sequences can find a copy in a PBM array, a structure with a binding site larger than 10 is avoided in the validation set. In total, there are 11 proteins (validation set 2) that satisfy all the aforementioned criteria, as shown in Supplementary Table S3.

Finally, to evaluate the performance of PiDNA in selecting real binding sequences from a set of random sequences, we used the binding sequences collected in the study by Morozov *et al.* (9) as positive samples and randomly generated 10 000 sequences as negative samples to construct validation set 3. This set includes 15 proteins.

### Comparison with annotated PFMs

**Figure 1.**
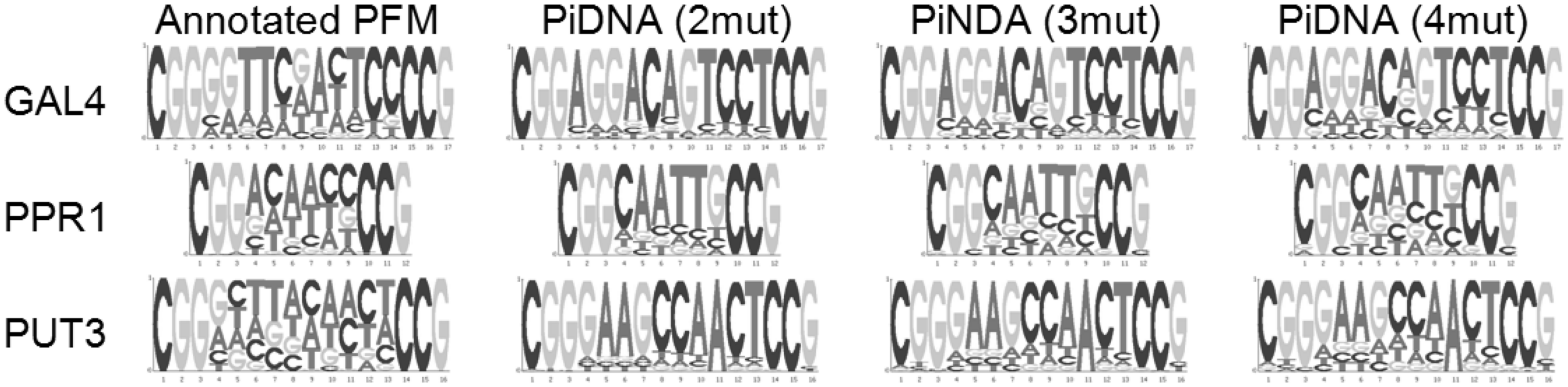
More mutations are desirable on binding sites with a large number of degenerated positions. The term ‘*k*mut’ denotes that the maximum number of mutations in a single sequence is set to *k* when constructing the PFMs.

The PFMs constructed by PiDNA, based on the set of favourable structure models with limited mutations, are compared with the annotated PFMs, and the performance is compared with the predictions by 3DTF and 3D-footprint. The *Ψ*-test described in Morozov *et al.* (9) was used to evaluate the consistency between the predicted and annotated weight scores. The definition of the *Ψ*-test is provided as follows:

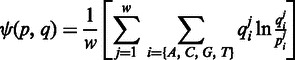

where 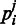 and 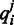 are predicted and annotated weight scores, respectively, for base type *i* at position *j*, and *w* is the length of the binding site in base pairs. A smaller value on the *Ψ*-test implies a higher degree of consistency between two PFMs. The range of mutated positions for each test case is shown in Supplementary Table S4, and the same range is applied to 3DTF. The results provided in Table 1 reveal that PiDNA is able to deliver PFMs similar to the annotated PFMs from databases or literature. Both PFMs from PiDNA and 3D-footprint are better than that constructed by the original sequence in the complex. It can be seen in Table 1 that, in general, setting the maximum number of mutations to two performs better than three and four. However, it is also observed that a larger number is preferred for binding sites with a large number of degenerated positions, as shown by the sequence logos plotted in Figure 1.

**Table 1.** Comparison of predicted PFMs with annotated PFMs based on the *Ψ*-test

PDB ID	Ori-seq	PiDNA-2mut	PiDNA-3mut	PiDNA-4mut	3DTF	3D-footprint
1aay	0.131	**0.087**	0.111	0.169	0.161	0.101
3dfv	0.184	**0.104**	0.141	0.193	0.619	0.122
2wty	0.421	**0.225**	0.246	0.271	0.706	0.558
1ig7	0.381	**0.168**	0.170	0.177	0.585	0.172
3u2b	0.621	0.461	0.461	0.461	0.965	**0.287**
3f27	0.388	0.129	0.129	0.129	0.878	**0.061**
1ysa	**0.173**	0.223	0.324	0.342	0.274	**0.173**
2er8	0.205	**0.123**	0.136	0.170	0.718	0.289
1mnn	0.182	**0.105**	0.119	0.123	0.158	0.151
1a0a	**0.172**	0.308	0.408	0.517	0.898	0.457
3ukg	0.713	0.475	0.430	**0.408**	0.713	0.486
3mln	0.364	0.245	**0.243**	**0.243**	0.603	0.389
1dh3	0.133	**0.132**	0.187	0.270	–^a^	0.321
1awc	0.134	0.139	0.251	0.270	–^a^	**0.126**
1puf	0.342	0.216	**0.206**	0.215	0.395	0.334
1h88	0.326	**0.139**	0.159	0.168	0.364	0.177
2ql2	0.372	0.179	0.205	0.205	0.529	**0.153**
3exj	0.353	**0.198**	**0.198**	**0.198**	0.290	0.296
1io4	**0.044**	0.162	0.261	0.261	0.440	0.045
3qsv	0.581	0.398	**0.384**	**0.384**	0.434	0.442
1gt0	0.307	0.168	**0.154**	0.157	0.566	0.167
1pue	0.205	**0.167**	0.185	0.215	0.355	0.194
3brg	**0.101**	0.135	0.196	0.282	0.833	0.134
2i9t	0.226	0.210	0.210	0.210	0.459	**0.178**
1d66^b^	0.773	0.441	0.355	**0.293**	0.412	–^c^
1yrn	0.293	**0.271**	0.302	0.319	0.496	0.300
1le8	0.226	**0.143**	0.162	0.208	0.499	0.511
1mnm	0.408	0.162	**0.154**	0.158	0.371	0.485
1pyi^b^	0.513	0.173	0.127	**0.104**	0.476	–^c^
1zme^b^	0.902	0.260	0.205	**0.165**	–^a^	0.297
Average	0.339	**0.212**	0.227	0.243	0.526	0.265
Standard deviation	0.209	0.107	0.098	0.100	0.214	0.147

A smaller number on the *Ψ*-test implies a higher degree of consistency between two PFMs.

‘Ori-seq’ denotes the PFM constructed by the original (native) sequence in the protein–DNA complex.

The title ‘PiDNA-*k*mut’ denotes that PiDNA constructed the PFM based on selected sequences with at most *k* mutations.

The best performance on each row is highlighted in bold.

^a^(3DTF) No prediction available.

^b^The sequence logos of the predicted PFMs are shown in Figure 1.

^c^(3D-footprint) No structural evidence for specific binding to DNA (<4 informative columns).

### Identification of binding sites with high specificity

Given a protein–DNA complex, it is of interest to know what other sequences with limited mutations from the native sequence can also be bound by the proteins in the complex. The expected relative binding specificity of these mutated sequences is retrieved from the *in vitro* PBM data. A 10-mer sequence can find two copies (including the reverse complementary form) in a PBM array constructed based on the *de Bruijn* sequence that covers all 10-mer binding sites. A site with shorter length can find more copies. It is assumed that the relative binding specificity of a *k*-mer string can be represented by the scores of the probes that contain the string. Because there is a risk that the binding specificity of the string of interest might be affected by other substrings on the same probe sequence, the average scores across all the probes that contain either the target string or its reverse complementary form are adopted. To evaluate the performance of PiDNA in detecting the binding sites with high specificity, the mutated sequences with relatively high binding specificity are assigned as the positive instances. The top-*k* scored sequences from PBM are adopted as the positives, where *k* is set to 10, 20, 50 and 100, respectively. Afterwards, the receiver operating characteristic (ROC) curve for each method is plotted based on the ranking list it produced.

**Figure 2.**
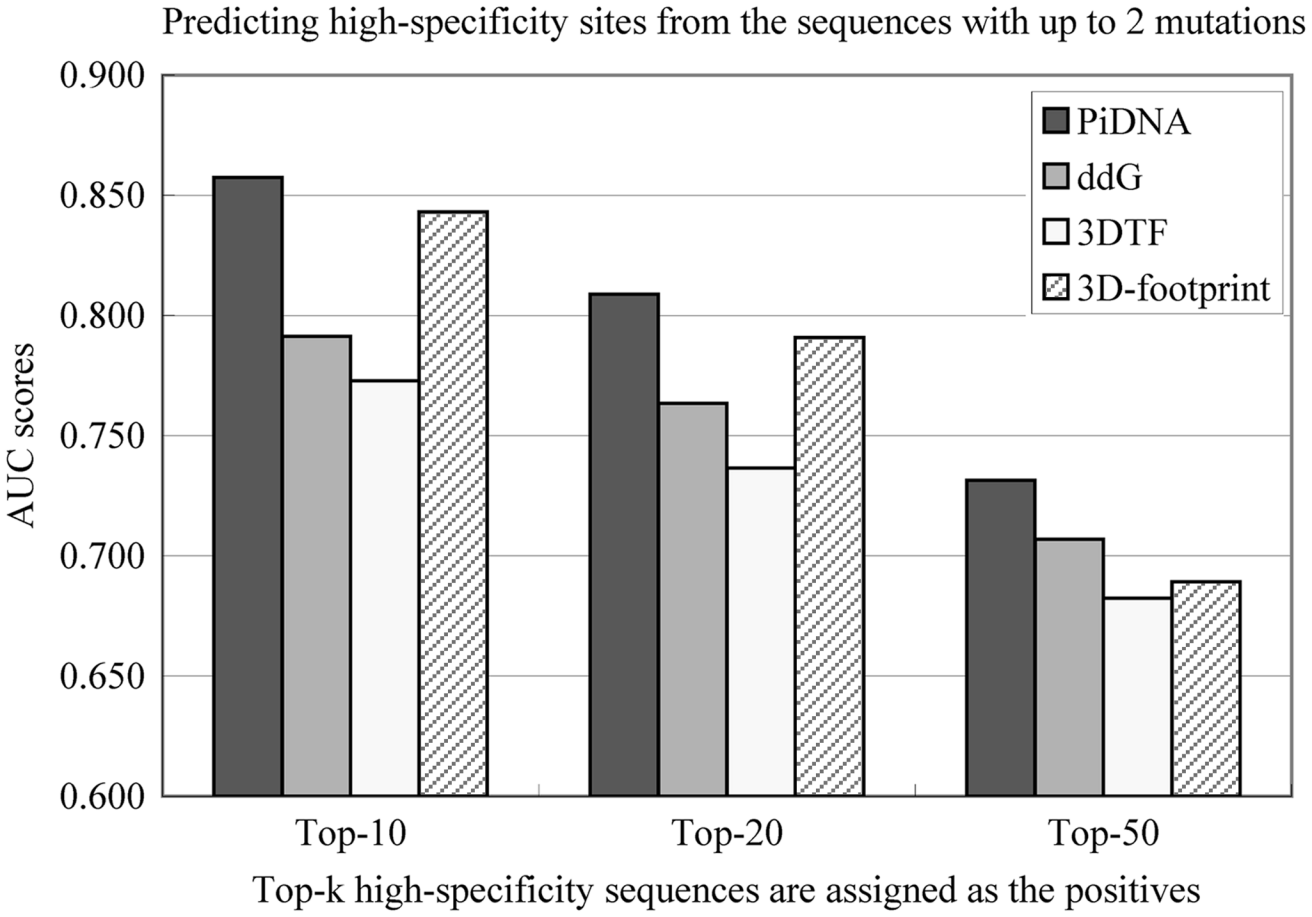
Comparison of PiDNA in predicting high-specificity sites among all the sequence with up to two mutations using the AUC scores. The method ‘ddG’ denotes the ranked list produced based on the change on Δ*G*, i.e. Δ*G*′ − Δ*G*_native_.

The testing data first include all sequences with up to two mutations. For the 11 proteins in validation set 2, the number of testing sequences ranges from 153 to 435. The ROC curves and the area under ROC (AUC) scores are calculated using the R package ‘ROCR’. The AUC scores of PiDNA are compared with the ranked lists generated by the PFMs from 3DTF and 3D-footprint. The same ranges shown in Supplementary Table S4 are applied when invoking the Web server 3DTF to generate PFMs for comparison. For 3DTF, the mode ‘long (for convergence of full model)’ is adopted. The average AUC scores for different methods are provided in Figure 2, and the AUC scores for each query protein are shown in Table 2 when the top-10 PBM scored sequences are used as positives. It is observed both in Figure 2 and Table 2 that PiDNA with PFM scoring is superior to the other approaches in identifying high-specificity sequences from the lower ones, including the approach of using the change on Δ*G* (ddG) for the ranking. This reveals that the mutated structure models and/or the selected atomic-level scoring function still have potential limitations.

**Table 2.** AUC scores for different Web servers based on validation set 2

Protein	PiDNA (PFM)	PiDNA (ddG)	3DTF	3D-footprint
Zif268 (mouse)	0.912	0.897	0.928	0.878
Gata3 (mouse)	0.932	0.842	0.738	0.944
Mafb (mouse)	0.775	0.798	0.669	0.821
Msx1 (mouse)	0.842	0.701	0.859	0.832
Sox4 (mouse)	0.915	0.933	0.825	0.938
Sox17 (mouse)	0.959	0.958	0.773	0.929
Gcn4 (yeast)	0.687	0.548	0.744	0.776
Leu3 (yeast)	0.713	0.498	0.659	0.709
Ndt80 (yeast)	0.933	0.905	0.886	0.870
Pho4 (yeast)	0.901	0.756	0.547	0.745
Rap1 (yeast)	0.863	0.870	0.872	0.832
Average	0.857	0.791	0.773	0.843

The testing data in this table include sequences with up to two mutations.

The top-10 high-specificity sequences are assigned as the positives.

The performance of PiDNA based on PFM scoring or based on the change on Δ*G* (denoted as ‘ddG’) is also compared.

Next, evaluation is performed on a larger testing test, i.e. sequences with up to four mutations from the native sequence. For the 11 proteins in validation set 2, the number of testing sequences ranges from 1909 to 20 686. In this analysis, PiDNA is executed with different settings on the maximal number of mutations allowed when constructing the PFMs. The results are shown in Supplementary Figure S2. Two observations are summarized here. First, it is observed that setting the maximal number of mutations to two when constructing PFMs performs better than setting it to three or four. This is consistent with the conclusion drawn from Table 1, the comparison of the predicted PFMs with the annotated ones. Second, it is shown again that PiDNA with PFM scoring is superior to the other approaches in identifying high-specificity sequences from the lower ones.

### Identification of known binding sites from random sequences

With validation set 3, PiDNA is evaluated according to its ability in identifying true binding sites from a set of 10 000 random sequences. The known binding sites are collected from the study of Morozov *et al.* (9), and length of the sites (the positions to mutate when constructing PFMs) is specified accordingly. 3D-footprint was not included in this comparison because most of the curated PFMs are shorter than the length of the sites for prediction. The results shown in Table 3 reveal that PiDNA can distinguish true binding sites from random ones with high accuracy. This data set includes 244 known binding sites, where 93 of the known sites contain more than four mutations with respect to the native sequences in the complexes used (Supplementary Figure S3). Table 3 reports the sensitivity (true-positive rate) and specificity (true-negative rate) for each query protein when high-specificity rates are considered, resulting in 28 false-negatives. It is summarized in Supplementary Figure S3 that most of the false-negatives are with a large number of mutations, although some sites with a large number of mutations can still be identified by PiDNA successfully. Instead of using sensitivity and specificity rates (there is a trade-off between them), the AUC scores are adopted when different methods are compared in Supplementary Table S5. Although the results in Supplementary Table S5 show that the change on Δ*G* alone does not serve as a good indicator for identifying true binding sites, it was observed that the change on Δ*G* usually assigns good rankings to the known binding sites among the sequences with the same number of mutations. In fact, the binding site with 12 mutations has a negative change on Δ*G*, and it is observed that more than half of the known binding sites are with a considerably small change on Δ*G*. Similarly, the RMSD values alone are not a good indicator, either. In PiDNA, RMSD values are reported along with the predictions such that users can be aware of large RMSDs, as the real conformational change might be even larger when the backbone flexibility is allowed.

**Table 3.** Data set (validation set 3) used for evaluating the performance of PiDNA in detecting true binding sequences from random sequences

Protein	Species	PDB	Number of sites	Width of the sites	True-positive rate (%)	True-negative rate (%)
Zif268	*Mus musculus*	1aay	6	10	100.00	99.98
Ndt80	*Saccharomyces cerevisiae*	1mnn	8	12	87.50	99.44
Gcn4	*S. cerevisiae*	1ysa	9	7	100.00	99.43
MAT a1/alpha2	*S. cerevisiae*	1yrn	19	19	100.00	99.09
EcR/Usp	*Drosophila melanogaster*	1r0o	33	15	87.88	99.86
Ttk	*D. melanogaster*	2drp	16	11	62.50	98.77
Prd (homeo)	*D. melanogaster*	1fjl	15	13	100.00	98.99
Ubx/Exd	*D. melanogaster*	1b8i	4	10	100.00	99.95
Trl	*D. melanogaster*	1yui	5	7	100.00	99.58
MetJ	*Escherichia coli*	1mj2	16	16	100.00	99.40
TrpR	*E. coli*	1tro	15	18	86.67	99.56
PhoB	*E. coli*	1gxp	16	20	93.75	99.32
DnaA	*E. coli*	1j1v	9	13	77.78	99.98
PurR	*E. coli*	2puc	23	16	100.00	99.90
Crp	*E. coli*	1run	50	9	76.00	98.46

Data are retrieved from the Supplement of Morozov *et al.*, 2005.

Testing data include the listed numbers of positives and 10 000 negatives.

## CONCLUSION

PiDNA is designed to predict PFMs of DNA-binding proteins based on available protein–DNA complexes. Numerous structure models are first generated with limited mutations. Afterwards, the mutated sequences with relatively small energy changes are used for constructing PFMs for more predictions. In this study, PiDNA is shown to achieve good performance in delivering reliable PFMs and discovering binding sites with high binding specificity among the set of sequences with limited mutations from the native structure. The analysis shown in this study also reveals that the PFMs reported by PiDNA are able to distinguish true binding sites from random ones with high accuracy. Therefore, PiDNA can be considered as an alternative approach to predicting binding sites of a TF in the genome when lacking well-characterized PFMs (or PWMs). With PiDNA, the users can design biological experiments based on the predicted sequence specificity and/or request mutated structure models for further protein design. Also, it is expected that PiDNA can integrate with other high-throughput data to refine large-scale inference of *in vivo* protein–DNA interactions.

## SUPPLEMENTARY DATA

Supplementary Data are available at NAR Online: Supplementary Tables 1–5 and Supplementary Figures 1–3.

## FUNDING

National Science Council of Republic of China, Taiwan [NSC 100-2627-B-002-002], and Center for Systems Biology, National Taiwan University. Funding for open access charge: National Science Council of Republic of China.

*Conflict of interest statement.* None declared.
